# Clinical effect and safety of medicated thread moxibustion on xanthelasma palpebrarum: A retrospective cohort study

**DOI:** 10.1097/MD.0000000000043223

**Published:** 2025-07-04

**Authors:** Lieling Kou, Weishai Liu, Xiaoyan Zhu, Wanyu Wan, Manhua Wang

**Affiliations:** a Department of Ophthalmology, Ankang Hospital of Traditional Chinese Medicine, Ankang, Shaanxi Province, China; b Shaanxi University of Chinese Medicine, Xianyang, Shaanxi Province, China.

**Keywords:** medicated thread moxibustion, safety, treatment sessions, xanthelasma palpebrarum

## Abstract

Medicated thread moxibustion (MTM), an integral component of traditional Chinese medicine, has, however, seen limited evidence regarding its application in treating xanthelasma palpebrarum (XP) in practical settings. This study was designed with the objective of evaluating both the efficacy and safety of MTM in the treatment of XP. A retrospective analysis was conducted on 79 patients with XP treated with MTM at a single center. The degree of lesion clearance, number of treatment sessions, adverse events, tolerability, and recurrence rate were assessed. By the final visit, 22 patients (28%) had achieved complete lesion clearance, while 54 patients (68%) exhibited significant improvement, with over 50% of their lesions cleared. Three patients (4.0%) experienced <50% clearance, indicating no significant therapeutic effect. The median number of treatment sessions required was 3.0, with an interquartile range of 2.0 to 5.0. Over half of the patients (51, 64.6%) achieved either excellent clearance or a satisfactory response within the initial 3 treatment sessions. Participants experienced tolerable itching or burning sensations, which obviated the need for anesthesia. No severe adverse events, including infection, atrophy, scarring, hyperpigmentation, or discoloration, were reported. However, in 3 patients (13.6%), lesions recurred within 1 year post-complete clearance. MTM suggests to be an effective and safe alternative for treating XP, featuring a low recurrence rate. Prospective randomized controlled trial studies are necessary to explore the mechanism and efficacy of this treatment method.

## 1. Introduction

Xanthelasma palpebrarum (XP) is the most common type of xanthoma, accounting for over 95% of cases. It is characterized by the deposition of lipid-rich material, primarily cholesterol, in the subcutaneous tissues. XP presents as yellowish plaques or elevated papules, often symmetrically distributed on the upper and medial canthus of the eyelids.^[1]^ The prevalence of XP varies across different populations, with estimates of 1.2% in China and 0.3% to 4% in European countries.^[2]^ It is more commonly seen in women and elderly individuals in their fourth and fifth decades.^[3]^ In addition to its cosmetic implications, patients with XP have an increased risk of developing atherosclerosis,^[4]^ metabolic syndrome,^[5]^ and nonalcoholic fatty liver disease.^[6]^

Although XP is usually benign, some cases may require treatment for cosmetic reasons or due to the possibility of enlargement. Several treatment options have been described in the literature, including surgical excision, laser therapy, chemical peels, and radiofrequency ablation (RFA). Surgical excision, with or without skin grafting, is the most commonly used method, but it carries the risk of complications such as ectropion and scarring.^[7]^ Other modalities such as chemical peels and laser therapy can result in skin discoloration, hyperpigmentation, and severe scarring.^[3]^ RFA is considered a safe but less effective option for treating XP.^[1]^

Medicated thread moxibustion (MTM), a therapeutic modality traditionally employed by the Zhuang ethnic group, has been utilized for the treatment of various dermatological conditions, including alopecia areata and psoriasis vulgaris.^[8,9]^ This technique involves the preparation of specialized ramie threads, which are immersed in a solution of traditional Chinese medicinal herbs and then subjected to direct moxibustion on specific acupoints or lesions of the human body post-ignition. MTM is characterized by its distinct thermal and concentrated thermal radiation effects, which facilitate the release of a multitude of pharmacological molecules during the combustion process. These molecules are rapidly adsorbed onto the body’s surface following high-temperature fumigation, creating a high-concentration pharmacological area in the vicinity of the epidermis and are transported throughout the body via the meridian system.^[8–12]^ Although MTM has been reported to be effective for XP during conferences, there are no studies evaluating its efficacy and safety for this condition. To gain a better understanding of MTM and its potential use in treating XP, this study aimed to comprehensively assess MTM for the treatment of XP in Chinese population.

## 2. Materials and methods

This retrospective cohort study included patients who attended a single academic medical center of Traditional Chinese Medicine (Ankang Hospital of Traditional Chinese Medicine) over a 5-year period and were diagnosed with XP. To avoid selection bias, all consecutive patients who met the predefined criteria during the study period from our center were included. Inclusion criteria were as follows: Patients with a clinical diagnosis of XP. No restrictions on age or gender. Patients without a history of photosensitivity, bleeding, or coagulation disorders. Patients who can attend regular follow-up visits and accept follow-up. Patients who had undergone surgical excision, laser therapy, or other treatments were also eligible for inclusion. Exclusion criteria were defined as follows: Patients with a follow-up period of <12 months were excluded. Patients with contraindications to MTM treatment, such as those with allergies to the ingredients used in the treatment. Pregnant or breastfeeding women, considering the potential risks of treatment to the fetus or infant. Patients with severe heart disease, liver or kidney dysfunction, or other systemic diseases that may affect the evaluation of treatment efficacy or safety.

**Figure 1.**
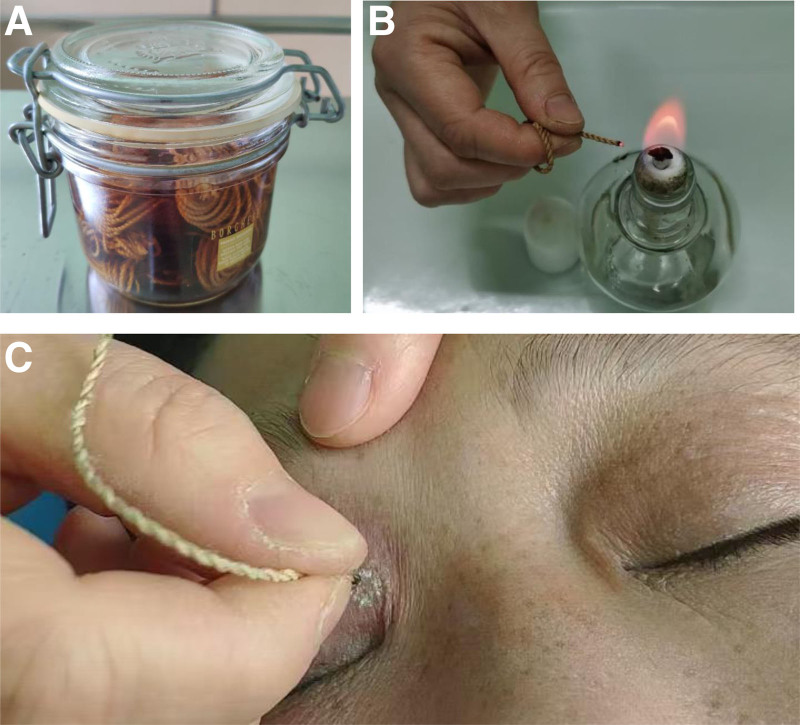
(A) About 0.25 mm diameter ramie threads, saturated in a proprietary medicinal solution, (B) the exposed segment of the thread was ignited, producing a bead-like spark, and (C) the ignited extremity of the thread was applied directly to the lesion for a duration of 2 s.

The MTM procedure involved the following steps:

1) The patient was positioned supine, with the lesion meticulously cleaned, eschewing the application of disinfectants or anesthetics.
2) A 0.25 mm diameter ramie thread, saturated in a proprietary medicinal solution, was selected (Fig. 1A).
3) The exposed segment of the thread was ignited, producing a bead-like spark (Fig. 1B).
4) The ignited extremity of the thread was applied directly to the lesion for a duration of 2 seconds (Fig. 1C).
5) Upon extinguishing the spark, the residual ash at the thread’s terminus was cleared, and the thread was rekindled to apply pressure to additional lesion areas.
6) Upon completion of the treatment across the entire lesion, the resultant ash was delicately removed with a sterile cotton swab.
7) The procedure was reiterated twice more.
8) Subsequent treatment sessions were scheduled at approximately 10-day intervals, contingent upon necessity.

The area of XP serves as a metric for evaluating and monitoring changes in lesion size, which is determined by calculating the product of length and width measurements obtained using a vernier caliper. The therapeutic effect of MTM was evaluated based on the clearance of the initial lesion area. Complete clearance (100%) was considered a cure, partial clearance (50–99%) was defined as improvement, and <50% clearance was classified as no effect. Treatment sessions were continued until the patient was satisfied or all lesions were resolved. The number of treatment sessions and any adverse events were documented. Patients were asked to assess the tolerability of MTM without anesthesia. Recurrence was defined as the discovery of a new lesion at the site of the original lesion.

Statistical analysis involved descriptive statistics calculations. Binary variables were expressed as frequencies and percentages, and quantitative variables were presented as median and interquartile range (IQR) or mean and standard deviation. The Shapiro–Wilk test was used to assess normality, and Spearman rank correlation analysis was performed to determine any correlation between age and the number of lesions.

The implementation of standardized training sessions, in conjunction with the utilization of predefined data collection forms, served to ensure consistency. Regular meetings were held to discuss any discrepancies and ensure adherence to the study protocol. This study was approved by the Ethics Committee of Ankang Hospital of Traditional Chinese Medicine (No. 20170801) and conducted in accordance with the Helsinki Declaration. All the patients involved in this manuscript have provided their written informed consent via a detailed consent form. This form comprehensively elucidates the purpose of the study, the procedures to be followed, potential risks and benefits, as well as the utilization of their de-identified, anonymized, and aggregated data and case details (including photographs) for publication purposes.

## 3. Results

Between June 2017 and May 2022, a total of 79 patients with XP were included in this retrospective cohort study conducted at our academic center. All patients were of Chinese ethnicity, with 18 males (22.8%) and 61 females (77.2%). The age of the patients ranged from 23 to 87 years, with a mean age of 51.1 (standard deviation 10.6) years. The XP lesions were either single or symmetrically located on the upper or lower eyelids of both eyes. Double lesions were the most common, observed in 59 patients (74.7%). There was no significant correlation between the patients’ age and the number of xanthelasma lesions (*P* = .772). The baseline demographic and clinical characteristics of the patients are summarized in Table 1.

**Table 1:** Baseline demographic and clinical characteristics.

Parameter	Patients (n = 79) or values
Sex	
Male	18 (22.8%)
Female	61 (77.2%)
Age (yr)	
Range	23–87
Mean ± SD	51.1 ± 10.6
Number of lesions	
Single	17 (21.5%)
Double	59 (74.7%)
Triple	0 (0%)
Quadruple	3 (3.8%)

SD = standard deviation.

At their final visit, 22 patients (28%) achieved complete clearance of their XP lesions, while 54 patients (68%) showed improvement with more than 50% clearance. Four lesions in 3 patients (4%) had <50% clearance and were considered to have had no effect.

The median number of treatment sessions required was 3.0 (IQR 2.0–5.0). More than half of the patients (51 patients, 64.6%) completed their treatment with excellent clearance of lesions or a satisfactory response within 3 treatment sessions. However, additional treatment sessions were necessary for patients who did not initially achieve satisfactory results. Further details regarding the treatment sessions are provided in Table 2.

**Table 2:** Treatment sessions of included patients.

Number of treatment sessions	Patients (n = 79) or values
1	18 (22.8%)
2	19 (24.1%)
3	14 (17.7%)
4	8 (10.1%)
5	10 (12.6%)
6	9 (11.4%)
7	1 (1.3%)
Median (IQR)	3.0 (2.0–5.0)

IQR = interquartile range.

**Figure 2.**
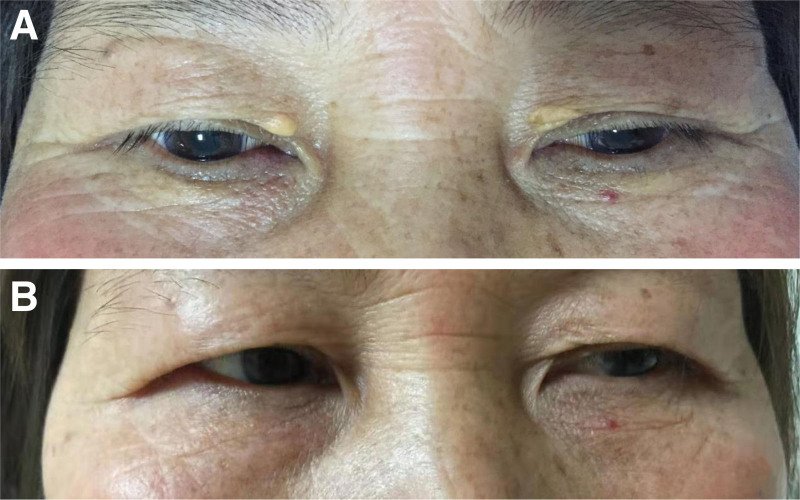
(A) A patient before treatment and (B) a patient after 1 treatment session.

**Figure 3.**
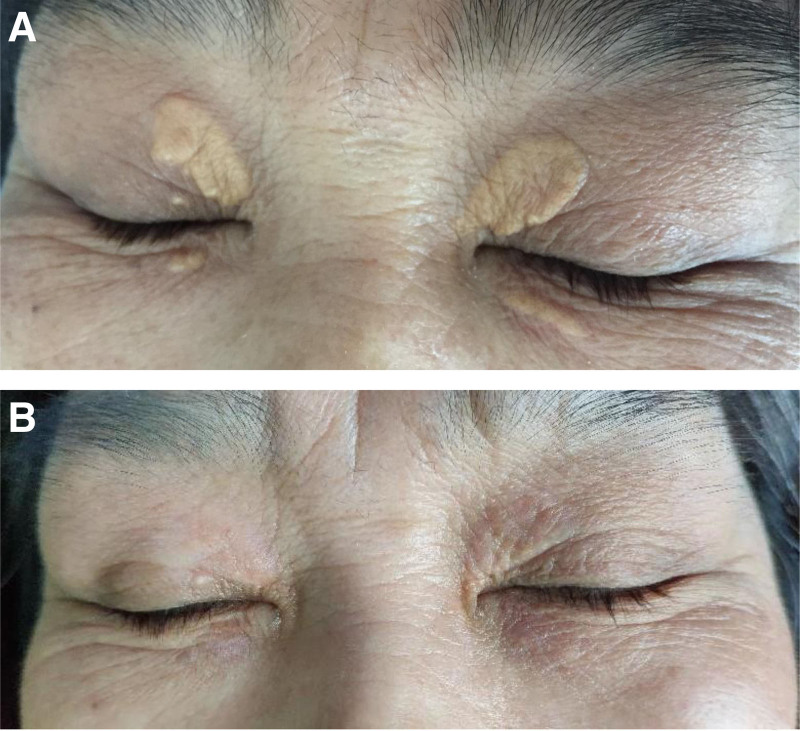
(A) A patient before treatment and (B) a patient after 3 treatment sessions.

Following the MTM procedure, the XP lesions transformed into reddish crusts surrounded by an annular erythema, accompanied by mild local itching or a burning sensation. The erythema typically resolved within 2 to 3 days, and the crusts peeled off approximately 1 week after treatment. Eventually, the color of the treated area gradually approached that of the surrounding normal skin within approximately 10 days. All participants reported tolerable itching or burning sensations and did not require anesthesia. Furthermore, no significant adverse events, such as infection, atrophy, scarring, hyperpigmentation, or discoloration, were observed in any of the cases after treatment. During the follow-up period, 3 lesions in 3 patients (13.6%) experienced recurrence 1 year after achieving complete clearance. Two examples of XP patients before and after MTM treatment are illustrated in Figures 2 and 3.

## 4. Discussion

In this retrospective cohort study, we investigated the efficacy and safety of MTM in the treatment of 79 Chinese patients with XP. Our findings suggest that MTM is a safe and effective treatment option with a low recurrence rate. Laser therapy is commonly used for treating XP, particularly when the lesions are superficial.^[13]^ However, its efficacy has been variable.^[3]^ Surgery, such as excision with or without grafts or flaps, is the conventional method for treating XP, particularly for lesions that involve deeper layers.^[14–16]^ Chemical peels have also been extensively studied and shown to be effective.^[17–19]^ It was found to be inferior to the use of carbon dioxide laser, which resulted in a clearance rate of 99.5%, as well as RFA, which resulted in a clearance rate of 98.8%. However, its clearance rate was found to be superior to that of topical trichloroacetic acid peel (92%), voltaic acid peel (89.4%), erbium yttrium aluminum garnet (87.8%), pulsed dye laser (75%), and Q-switched neodymium-doped yttrium aluminum garnet (71.2%).^[3]^ In comparison to these modalities, MTM demonstrated an improvement or clearance rate higher than 50% in 96% of cases in our study, making it comparable in efficacy.

MTM is a minimally invasive procedure that can be performed without anesthesia, which distinguishes it from other treatment modalities. Multiple treatment sessions may be required, as seen with other treatment options such as laser therapy and chemical peels.^[17,20]^ The median number of treatment sessions required in the current study was 3.0 (IQR 2.0–5.0). Chemical peels and RFA have been previously shown to yield satisfactory results, particularly when multiple treatment sessions are performed. However, the existing literature does not provide specific details regarding the exact number of treatment sessions required.^[17,21]^ Nevertheless, a study investigating the effects of 1444 nm neodymium-doped yttrium aluminum garnet laser treatment reported a mean of 1.9 treatment sessions, with a standard deviation of 1.2, indicating a potential advantage of this approach.^[20]^ However, further research is needed to determine which treatment method necessitates the fewest sessions.

Recurrence is a concern in XP treatment, regardless of the modality used. Underlying conditions like hyperlipidemia may also contribute to recurrence.^[1,22]^ Surgical techniques have reported recurrence rates ranging from 3% to 28%,^[15,23,24]^ while laser therapy has shown rates ranging from 9% to 48%.^[25–27]^ Chemical peels have reported recurrence rates of 5% to 32%.^[17,18,28]^ In our study, the recurrence rate was 13.4%, which aligns with the high recurrence rates observed across treatment methods.^[1]^

Each treatment modality for XP has specific complications and limitations. Continuous mode carbon dioxide laser therapy carries the risk of scarring and dyspigmentation as its penetration is unpredictable.^[29]^ Chemical peels can be limited by side effects such as hypo- or hyperpigmentation, scarring, and atrophy.^[19]^ In addition, it has also been reported to cause a Koebner-like phenomenon.^[30]^ Surgery, while invasive, can result in adverse events like dyspigmentation, lid deformity, or scarring.^[3]^ MTM, on the other hand, is a low-risk procedure associated with no major complications, making it advantageous over other modalities.

MTM is predicated on the mechanism of medicated thread combustion, which liberates drug molecules for absorption by the body and subsequent topical action.^[9]^ Nevertheless, the precise underpinning mechanism remains elusive, warranting additional investigation to delineate the contributions of both the thread and the medicament. To the best of our knowledge, this study represents the pioneering exploration of MTM in the treatment of XP. It demonstrates comparable efficacy to alternative treatment modalities, while also being well-tolerated and associated with minimal complications. MTM merits consideration as a viable treatment option, particularly in regions where access to laser equipment is limited.

Limitations of our study include the absence of a control group, which limits the understanding of the relative efficacy of different medicated threads or liquid medicines. Prospective randomized controlled trial studies are necessary to explore the mechanism and efficacy of this treatment method. Additionally, the short follow-up period in our study may have underestimated the recurrence rate, highlighting the need for longer follow-up in future studies. Moreover, it is recognized that the findings of our study are primarily derived from a Chinese population. When the generalizability of our findings is considered, cultural and healthcare system differences need to be taken into account. In Chinese culture, there may be differences in patient compliance, family support, and beliefs about treatment. The healthcare system also has unique features such as access to certain medications and the organization of healthcare delivery. In future research, collaborations with international teams should be considered to conduct multicenter studies in different countries to assess the generalizability of MTM across different populations. Simultaneously, efforts should be made to boost patients’ confidence in undergoing MTM treatment, ensuring the seamless completion of the entire treatment course. Such measures will enable a more in-depth and comprehensive understanding and acceptance of this treatment approach across a broader spectrum.

## 5. Conclusions

MTM suggests to be an effective and safe alternative for treating XP, featuring a low recurrence rate. Prospective randomized controlled trial studies are necessary to explore the mechanism and efficacy of this treatment method.

## Acknowledgments

The authors are deeply appreciative of the financial support provided by the Shaanxi Administration of Traditional Chinese Medicine.

## Author contributions

**Data curation:** Wanyu Wan.

**Investigation:** Xiaoyan Zhu.

**Methodology:** Wanyu Wan.

**Validation:** Manhua Wang.

**Writing – review & editing:** Lieling Kou.

**Writing – original draft:** Weishai Liu.
